# Inborn errors of immunity in adulthood

**DOI:** 10.1186/s13223-023-00862-8

**Published:** 2024-01-17

**Authors:** Joanne J. F. Wang, Arün Dhir, Kyla J. Hildebrand, Stuart E. Turvey, Robert Schellenberg, Luke Y. C. Chen, Persia Pourshahnazari, Catherine M. Biggs

**Affiliations:** 1 Department of Medicine, University of British Columbia, Vancouver, Canada; 2 Department of Pediatrics, BC Children’s Hospital Research Institute, University of British Columbia, Vancouver, Canada

**Keywords:** Inborn error of immunity, Primary immunodeficiency, Adulthood, Evaluation, Diagnosis

## Abstract

Inborn errors of immunity (IEIs) are a group of conditions whereby parts of the immune system are missing or dysfunctional. Once thought to primarily be a pediatric disorder, it is now estimated that more than 50% of worldwide incident IEI cases are accounted for by adults. Delayed diagnosis, late symptom onset, and IEI phenocopies can all lead to adult-onset recognition of IEIs. Lack of awareness regarding the diversity of IEI manifestations in adults contributes to diagnostic and treatment delays. Prompt referral to immunology is critical so that patients can receive a precise molecular diagnosis and targeted therapy when available. This article serves as a primer on IEIs in adulthood, highlighting the pathophysiology, epidemiology and clinical features. We present clinical vignettes of three key IEIs to assist clinicians in building illness scripts on their presentations. We provide a framework for the laboratory evaluation of IEIs and their initial treatment, with the aim of improving recognition and management of these conditions.

**Supplementary Information:**

The online version contains supplementary material available at 10.1186/s13223-023-00862-8.

## Background

Inborn errors of immunity (IEIs) are a group of inherited conditions in which parts of the human immune system are missing or dysfunctional [1]. The previously coined term “primary immunodeficiencies” has been recently replaced with IEIs in recognition of the diversity of clinical presentations, ranging from not only immune deficiencies, but also exaggerated or dysfunctional immune responses [2]. Clinically, IEIs lead to recurrent infections, malignancy, and immune dysregulation—a term referring to when the body’s immune response is uncontrolled, leading to inflammation and organ damage.

IEIs affect both children and adults, with more than 50% of worldwide IEI incident cases accounted for by those over 25 years-of-age [3]. IEIs are not rare, with estimated prevalence rates as high as 1:1200 [4], and are associated with significant morbidity and mortality [5]. Many clinicians have limited appreciation for IEIs as knowledge quickly becomes outdated in this fast-changing field. To date, there are more than 480 unique IEIs now described [6].

Unfortunately, adults with IEIs face significant diagnostic and treatment delays [7]. It is imperative that IEIs are recognized given the potential impact on management. Improved understanding of the specific genetic causes of IEIs has created the opportunity for targeted therapies. This precision-based approach can dramatically improve outcomes for patients by addressing the disrupted molecular pathway underlying the specific IEI [8]. However, before patients can receive personalized and potentially life-saving therapy, they need to be identified.

This article provides a primer on IEIs in adults, reviewing the pathophysiology, key clinical features and presentations, and outlines an investigative approach to an adult with suspected immunodeficiency.

### Pathophysiology

The immune system is a sophisticated network designed to tolerate self-antigens and non-pathogenic organisms while eliminating pathogens and infected or altered cells. To maintain human health, the immune system faces a formidable challenge: protect against infection and malignancy while preventing tissue injury caused by excessive inflammation and immune dysregulation. Humans have therefore evolved a multi-tiered system of immune defense, ranging from anatomical and physical barriers such as the skin, to cells and proteins with roles in early and late phases of the immune response.

These components of the immune system work together to maintain health, and a defect in any layer leads to compromised immunity. The international union of immunological societies has developed a comprehensive system for classifying the more than 480 unique forms of IEIs that exist [6]. However, in a more simplified view, IEIs can be broadly classified into disorders of innate immunity, adaptive immunity, and immune regulation.

The innate immune system is capable of rapid responses to eliminate pathogens. IEIs caused by genetic variants in innate signaling pathways, phagocytic cells or complement proteins are all considered innate immune defects. Complement defects are associated with bacterial infections and autoimmunity, while phagocytic defects primarily predispose to bacterial and fungal infections and immune dysregulation [9, 10]. Innate signaling defects can lead to either viral, bacterial, or fungal infections depending on the specific signaling pathway affected. IEIs caused by aberrant production of inflammatory cytokines lead to ‘autoinflammatory’ syndromes, characterized by recurrent fevers and organ system inflammation [11].

Adaptive immunity is a slower and more specific immune response that creates immunological memory and is formed by T-cells and B-cells. T-cells play an essential role in immunity: they destroy cancerous or virally infected cells, prevent immune dysregulation, and help activate other cells of the immune system such as B-cells and macrophages. The term “combined” immune deficiency thus refers to defects in T-cells, as an impaired T-cell response confers an associated defect in B-cells [12]. Disorders of B-cell development or antibody production predispose primarily to bacterial infections, however can also be associated with granulomatous inflammation, autoimmunity, lymphoproliferation and malignancy. Common variable immunodeficiency (CVID) is a form of IEI characterised by decreased serum immunoglobulin levels and impaired specific antibody production [5].

Equally important as initiating an immune response is the ability to effectively control the instigating trigger and the response that ensues. A dysregulated immune system can cause abnormal immune cell activation or proliferation, leading to cytopenias, solid organ autoimmunity, lymphoproliferative disorders, or malignancies [13]. Familial hemophagocytic lymphohistiocytosis (fHLH) is one example of an immune regulatory disorder, whereby impaired cytotoxic cell regulation and activity results in uncontrolled immune activation and life-threatening cytokine storm. While fHLH typically presents in childhood, secondary forms of HLH are more common in adults, whereby the hyperinflammation is driven by an underlying disease process such as lymphoma, autoimmune disease, or certain viral infections [14, 15].

### Clinical presentation

Box 1 provides clinical vignettes of three key IEIs to assist clinicians in building illness scripts surrounding IEI presentations. Several clinical scenarios can lead to an IEI being diagnosed in adulthood, including diagnostic delays, delayed clinical presentation, and phenocopies of IEIs. Though not considered an IEI, secondary immunodeficiencies (SIDs) are another cause of adult-onset immune deficiency. SIDs, also known as acquired immunodeficiencies, are caused by environmental factors. Examples include: viral infections such as human immunodeficiency virus (HIV), nutrient deficiency or hypoproteinemia leading to hypogammaglobulinemia, burns that compromise host defense conferred by epithelial barriers, and medications such as chemotherapeutics, biologics (ex: B-cell depleting agents such as rituximab), and small molecules (ex: Janus kinase inhibitors).

**Table:** 

Box 1: Clinical vignettes
**Vignette 1**
A 50-year-old male presents with a history of left leg lymphedema, basal cell carcinoma, and granulomatous uveitis. He has had recurrent sinopulmonary infections since young adulthood and experienced an atypical mycobacterial skin infection at the age of 30. Over the past three years, he has developed progressively worsening respiratory symptoms, including dyspnea and cough. A CBC with differential shows monocytopenia. He undergoes pulmonary function tests, which reveal a restrictive pattern with reduced diffusion capacity. A CT chest scan demonstrates a crazy-paving pattern, and bronchoscopy confirms a diagnosis of pulmonary alveolar proteinosis. The patient is referred to immunology, where they arrange immunophenotyping, revealing a deficiency in B and NK cells but normal T-cell numbers. Genetic testing in the form of a targeted inborn error of immunity gene panel identifies a heterozygous pathogenic variant in *GATA2*, consistent with GATA2 deficiency. He is then referred to hematology/oncology, and a bone marrow biopsy reveals evidence of trilineage myelodysplasia. The patient undergoes an allogeneic hematopoietic stem cell transplantation, resulting in normalization of hematologic and immune cell parameters and improvement in pulmonary alveolar proteinosis.
**Vignette 2**
A 25-year-old male presents with fever, cough, and difficulty breathing. He has a history of eczema with recurrent staphylococcal skin superinfections and pneumonias since childhood. He also experiences chronic back pain following a fall from standing one year prior. Physical examination reveals a broad nasal bridge, eczema, bilateral crackles, and diffuse wheezing upon lung auscultation, as well as tenderness upon palpation of the thoracolumbar spine. Laboratory studies show a mild eosinophilia and mildly elevated C-reactive protein. Sputum culture yields pseudomonas aeruginosa growth. The patient has normal levels of IgG, IgA, and IgM, but severely elevated IgE. A chest radiograph reveals a large pulmonary cyst in the left upper lobe, and a CT spine demonstrates vertebral compression fractures. Immunology is consulted and performs a targeted inborn error of immunity gene panel, which shows a heterozygous pathogenic loss-of-function variant in *STAT3*, consistent with the diagnosis of STAT3 deficiency, also known as autosomal dominant hyper IgE syndrome. He is initiated on sulfamethoxazole-trimethoprim and itraconazole for antimicrobial prophylaxis.
**Vignette 3**
A 31-year-old female presents with type 1 diabetes, a prior episode of immune thrombocytopenia, and enteropathy diagnosed as inflammatory bowel disease (IBD). Her IBD has responded poorly to tumor necrosis factor alpha inhibition. Further history reveals one prior episode of pneumonia treated with oral antibiotics. A CBC with differential shows neutropenia (an absolute neutrophil count of 0.8 × 10^9/L). After undergoing a bone marrow biopsy and having other causes of neutropenia excluded, she is diagnosed with immune-mediated neutropenia. Immunoglobulin levels indicate normal IgG, IgA and IgM levels. Additionally, she has protective vaccine titers to tetanus and diphtheria. The patient is referred to immunology, who arrange immunophenotyping that reveals normal absolute numbers of T, B, and NK cells; however, an elevated soluble IL-2 receptor level, in keeping with T-cell activation. Genetic testing in the form of a targeted inborn error of immunity gene panel identifies a heterozygous pathogenic variant in *CTLA4*, consistent with a diagnosis of CTLA-4 insufficiency. She is transitioned off tumor necrosis alpha inhibition and initiates targeted therapy with abatacept, a CTLA-4 fusion protein currently approved for the treatment of rheumatoid arthritis. Her enteropathy improves, and her neutrophil count and soluble IL-2 receptor levels normalize.

Diagnostic delays are an important cause of IEIs presenting in adulthood. This is defined as the time between onset of IEI symptoms to the time of diagnosis. Delays may represent the public and physician’s lack of awareness of IEI, as well as barriers to accessing technology needed to make appropriate diagnoses. A study from Vancouver, Canada found a mean latency of 16.1 years between symptom onset and the age at genetic diagnosis [16]. However, it is also possible that IEI manifestations may not occur until adulthood. For example, CVID epidemiological evidence suggests there may be two peaks of diagnosis, one during childhood prior to age 10, and the second during adulthood peaking at the third to fourth decade of life [17, 18]. Clinical vignette 1 in Box 1 describes a patient with GATA2 deficiency whose symptoms of immunodeficiency with recurrent infections did not occur until young adulthood. This is in contrast to Vignette 2, where a STAT3-deficient patient had recurrent infections in childhood but IEI was not considered by providers until the patient reached adulthood.

Late onset of symptoms can also be caused by hypomorphic variants in IEI genes, resulting in milder symptomatology and later presentation in life [19]. The term “hypomorphic” refers to genetic variants that alter protein function but retain sufficient activity that the immune defect is milder and may not manifest until later in life. For example, X-linked chronic granulomatous disease (CGD) classically presents in early childhood with severe manifestations, however, presentations later in childhood and even into late adulthood have been reported due to variants causing stable protein expression with hypomorphic function [20]. X-linked carriers of null variants can also develop disease later in life [19]. Progressive skewing of X-chromosome inactivation in hematopoietic stem cells overtime can result in progressive reduction of normal protein production, eventually leading to symptomatology [21]. Another mechanistic cause of IEIs presenting in adulthood is the effect of genetic redundancy whereby other immune-related genes compensate for the defective protein until later in life [22, 23]. Additionally, medical advances such as vaccination and prompt use of antibiotics in childhood may preclude the development of serious bacterial infections until the affected individual reaches adulthood, despite the person harbouring an immune defect.

“Phenocopies” of IEIs typically present in adulthood with clinical manifestations of an IEI, however are not caused by a genetic condition present since birth. Rather, IEI phenocopies are caused by either somatic (ie non-germline) variants that impact immune-related genes, or by autoantibodies targeting immune factors [24]. There are a number of unique IEI phenocopies caused by autoantibodies targeting cytokines, which are soluble proteins involved in host defense. These autoantibodies can develop in otherwise healthy individuals in an analogous manner to other autoimmune conditions. Anti-cytokine autoantibodies neutralise and inhibit the cytokine’s function thus causing specific immune defects related to the particular cytokine’s role in host defense. One example of this is neutralizing antibodies against type 1 interferons, which have been identified in approximately 10% of individuals with life-threatening SARS-CoV-2 infection [25].

Heterogeneous and diverse IEI presentations impel clinicians to maintain a low threshold for considering IEIs as a diagnostic possibility. Overall, IEIs have a male predominance of 1.5 to 1 (male:female ratio) [26]. Appreciating that adult IEIs present distinctly from IEIs in childhood, 10 warning signs for IEIs in adulthood were established, with the recommendation that two or more warning signs should prompt referral to a clinical immunologist [27]. This criterion was first developed in 1993 by the Jeffrey Modell Foundation (JMF) and has contributed greatly to public awareness [28]. Applying the criteria retrospectively to a cohort of 400 adults with IEIs found that less than 45% had two or more warning signs [29]. This study highlights that warning signs can help increase suspicion for IEIs, but should not be relied upon to identify all cases of IEIs given the heterogeneous manifestations of these conditions. Clinical phenotypes often extend beyond infectious complications (Box 1). A single-center study on 473 patients with CVID, the most prevalent clinically-significant IEI, followed over four decades found that 94% of patients had a history of infections, with 68% also suffering from non-infectious complications, including autoimmunity, chronic lung disease, gastrointestinal inflammatory disease, malabsorption, and malignancy [5].

### Assessment and diagnosis

Assessing for IEIs in adults should include a thorough review of the patient’s history of infections and immune dysregulation. Features of immune dysregulation can be subcategorized mechanistically as: (i) autoimmunity: a term used to describe adaptive immune responses against self-organs; (ii) autoinflammation: whereby dysregulated innate immunity causes fever and organ system inflammation; (iii) granulomatous inflammation; (iv) severe atopy: severe/atypical or refractory allergic conditions, or severe elevations in eosinophils and/or IgE; (v) lymphoproliferation, and (vi) malignancy: most frequently viral-driven- (ex: Epstein Barr virus or human papillomavirus) or blood-cancers (such as lymphoma), as well as cancers caused by excessive or prolonged inflammation, such as gastric malignancy associated with autoimmune gastritis [30–33]. Inquiring about the response to treatment of immune dysregulation can also help gauge ones’ level of suspicion for an underlying IEI. In clinical vignette 3 (Box 1), the patient had a limited history of infection, however also had multiorgan autoimmune disease including treatment-refractory inflammatory bowel disease, prompting an evaluation for IEI. People with IEIs may not necessarily have more severe infections, rather, they may experience recurrent or chronic infections from common pathogens resulting in end-organ damage [1]. Therefore, one’s infectious history should thoroughly characterise prior infecting pathogens, treatment response, related hospitalizations, and complications experienced. Clinicians should consider IEI for patients who experience severe or recurrent infections, respond poorly to antimicrobial therapy, or encounter opportunistic infections.

Past medical history, a thorough review of systems, as well as family history of IEI, consanguinity, or childhood deaths should be documented (Additional file 1: Table S1) [34]. In clinical vignette 2, the patient has a history of recurrent infections, atopy, chronic lung disease, and a prior minimal-trauma fracture. The fracture history is relevant and helps to further hone the differential diagnosis onto IEIs associated with connective tissue abnormalities, such as STAT3 loss-of-function. Additionally, features on history aimed at identifying potential secondary causes of immunodeficiency should be ascertained, such as nutritional and HIV status, and medication history including immunosuppressant use. A complete physical exam should be performed using a systems approach. The patient’s anthropometrics, presence or absence of lymphadenopathy, tonsils, and hepatosplenomegaly should be documented. Clinicians should assess for signs of infection or rheumatological disease including a thorough musculoskeletal and dermatological examination.

There are several laboratory investigations clinicians can use to further work-up suspected IEI (Table 1). Baseline infectious disease screening including testing for HIV should be obtained. Routine complete blood count and differential along with a peripheral blood smear can provide information on suspected cytopenias or gross hematologic abnormalities. Lymphopenia is an important indicator of a potential T-cell (cellular) immunodeficiency [28]. Inflammatory markers such as CRP are useful when assessing for inflammation and a patient’s ability to mount inflammatory responses. CH50 and AH50 assays evaluate the classical and alternative complement pathways, respectively, however flow cytometric tests assessing complement levels and function are also available clinically. Serum immunoglobulins (IgG, IgM, IgA, IgE) should be ordered to evaluate for B-cell disorders [34]. This can be useful in evaluating quantitative Ig deficiencies, such as X-linked agammaglobulinemia, CVID, or selective IgA deficiency, and humoral changes associated with other defects, such as hyper-IgE or hyper-IgM syndromes. Assessing serum-specific antibody titers in response to vaccine antigens such as tetanus or diphtheria toxoids is another critical diagnostic parameter when evaluating for antibody deficiency [28].

**Table 1:** Laboratory evaluation of inborn errors of immunity

Basic screening	HIV testing Complete blood count & differential, peripheral blood smear Renal and liver function Inflammatory markers
Innate immunity	Neutrophil functional assays C2, C3, C4 levels Complement activity assays (ex: CH50 and AH50 assays)
Adaptive immunity	T-cell/combined immunodeficiency: • Flow cytometry for B-, T-, NK-cell enumeration • T-cell memory panel • Lymphocyte functional assays B-Cell/predominately antibody deficiency: • Serum immunoglobulins: IgG, IgM, IgA, IgE • B-cell memory panel • Serum-specific antibody titers to vaccine antigens: tetanus, diphtheria, pneumococcus
Advanced testing	Anti-cytokine antibody assays Cytokine and cytokine receptor levels Cytotoxic cell protein expression & functional assays Genetic testing

While baseline immune studies can help further ones’ diagnostic suspicion for an IEI or narrow the differential diagnosis, certain IEIs have normal screening immune labs and rely on genetic testing for clinical diagnosis. It is therefore imperative that all patients in whom there is a clinical suspicion for an IEI be referred to an immunologist, even in the presence of normal screening labs. The immunologist can then perform more detailed immunophenotyping and genetic testing as indicated. Flow cytometric assays can be used to quantify lymphocyte subsets (B- T-, and NK-cells), evaluate markers of T- and B-cell maturation, autoreactivity and exhaustion, perform lymphocyte functional assays, and assess for expression of specific proteins that are deficient in particular IEIs [28]. For example, the neutrophil oxidative burst test is a functional assay performed by flow cytometry that can be used to detect CGD.

Genetic testing has become an integral part of the evaluation for an underlying IEI, given that most IEIs are derived from single gene defects. Technological advancements and improved access to genetic testing over the past two decades has rapidly expanded the number of genetically unique IEIs. This has been greatly facilitated by next generation sequencing, a technique that rapidly analyzes multiple segments of DNA simultaneously. Next generation sequencing powers many of the genetic tests in clinical use, including: i) targeted gene panels (TGP): sequencing of several genes at once that are relevant to the disease process of interest; ii) whole exome sequencing (WES): sequencing of exons/protein-coding regions of > 90% of known genes; and iii) whole genome sequencing (WGS): sequencing of the entire genome, including coding and non-coding regions [35]. Targeted gene panels are often easier to access and less costly than WES or WGS, however, require frequent updating of newly discovered disease-causing genes. This disadvantage is overcome by broader tests such as WES and WGS, however accessing these tests may be difficult [35].

### Management

Screening for complications related to IEI should be assessed during each clinician visit. The American Academy of Allergy, Asthma & Immunology (AAAAI) and the American College of Allergy, Asthma & Immunology (ACAAI) have jointly established practice recommendations when taking care of IEI patients which has been summarized in brief below [36]. Patients with IEI are at increased risk of developing malignancies compared to the general population [37]. From registry-based studies, the relative risk varies from 1.4 to 5-fold [38–41]. Therefore, clinicians should be aware of diligent age-appropriate or in certain IEIs enhanced malignancy screening in these patients. Routine blood cell count monitoring should be performed given the possibility of cytopenias or hematologic malignancies. Additional respiratory screening with routine lung imaging and lung function testing should be performed. This should be dictated by the patient’s particular IEI and clinical situation.

Primary prevention and prophylaxis helps to prevent serious infections and resulting morbidity and mortality. In certain IEIs associated with susceptibility to viral or bacterial infections, live vaccines can lead to severe complications [42]. The suitability of live vaccines (such as BCG, measles, mumps, rubella, and varicella) depends on the particular IEI, and clinicians should refer to specific published guidelines to help guide these decisions [43]. Immunoprophylaxis with immunoglobulin replacement therapy is indicated in all individuals who have severely impaired antibody production [36]. The use of antimicrobial prophylaxis depends on the particular defect conferred by the IEI and resulting pathogenic susceptibilities, and may include antibiotic, antifungal, or antiviral prophylaxis. For example, both CGD and STAT3 loss-of-function predispose to serious fungal and bacterial infections, therefore these conditions typically warrant regular use of both antifungal and antibacterial prophylaxis [44]. In conditions with a high risk of bronchiectasis secondary to recurrent bacterial infections or when bronchiectasis has already been established, antimicrobial prophylaxis should also be considered. When treating active infections, people with IEIs often require broad-spectrum antibiotics and long treatment durations, as standard antimicrobial dose and duration may not be adequate to eradicate infections in this population [44].

Until recently, the mainstay of IEI management included prompt infection treatment, antimicrobial prophylaxis, and immunoglobulin replacement. While these remain important management tools, the advent of precision-based therapies has dramatically enhanced the treatment armamentarium [35]. Understanding the molecular basis of IEIs facilitates the use of targeted therapies that can replace the missing or dysfunctional protein, calming immune dysregulation and restoring homeostasis [45]. These targeted therapies have been particularly useful in improving outcomes for inflammatory complications associated with IEIs [46] (Box 1). Allogeneic hematopoietic stem cell transplantation (HSCT) is a potentially curative therapeutic option for adults with certain IEIs, and involves replacing the individual’s immune system with a healthy donor’s. Concerns over potential risks of transplant-related mortality in adults has hampered adoption of HSCT for those with IEIs warranting transplant later in life [47]. A recent study on 329 adults with IEIs who underwent HSCT, however, has shifted that paradigm, providing evidence that HSCT is feasible and potentially curative in this population [47, 48]. While the study identified risk factors for adverse outcomes in HSCT, interestingly age had no significant impact on overall- or event-free survival [47].

## Conclusions

IEIs represent a group of conditions that clinicians will encounter in their careers. Early identification of IEIs remains a challenge given the wide array of manifestations people may present with. As these conditions lead to multi-organ complications, generalists as well as specialists across fields can play an important role in recognizing these disorders and initiating appropriate referral to a clinical immunologist. Increased awareness among clinicians combined with improved diagnostic and treatment options brings hope for a brighter future for people living with IEIs.

### Supplementary Information

**Additional file 1: Table S1.** Systems approach to IEI manifestations.

## Data Availability

Not applicable.

## Abbreviations

AAAAI: American Academy of Allergy, Asthma and Immunology
ACAAI: American College of Allergy, Asthma and Immunology
ASID: Acquired immunodeficiencies
CGD: Chronic granulomatous disease
CVID: Common variable immunodeficiency
fHLH: Familial hemophagocytic lymphohistiocytosis
HIV: Human immunodeficiency virus
HSCT: Hematopoietic stem cell transplantation
IEI: Inborn errors of immunity
SID: Secondary immunodeficiencies
TGP: Targeted gene panels
WES: Whole exome sequencing
WGS: Whole genome sequencing
