# Supplementary material for: Inborn errors of immunity in adulthood
Source: Allergy Asthma Clin Immunol. 2024 Jan 17;20:6. doi: 10.1186/s13223-023-00862-8 (PMC10792788; doi:10.1186/s13223-023-00862-8)
Supplement: Supplementary file 1 — Additional file 1: Table S1. Systems approach to IEI manifestations. [file 13223_2023_862_MOESM1_ESM.pdf]

Supplemental Materials

Table S1. Systems approach to IEI manifestations.

**System**	**Examples of presentations**
Pulmonary	Respiratory infections, interstitial lung disease, bronchiolitis obliterans, pulmonary alveolar proteinosis, recurrent serositis
Dermatologic	Infectious skin findings, erythroderma, eczematous lesions, cutaneous granuloma, vitiligo, psoriasis, photosensitive malar rash
Gastrointestinal	Chronic diarrhea, malabsorption, eosinophilic esophagitis, gastritis, inflammatory bowel disease, enteropathy, celiac-like disease, hepatitis, hepatomegaly, cirrhosis, cholangitis, biliary fibrosis, malignancies
Rheumatological	Vasculitis, arthritis, uveitis, systemic lupus erythematosus-like syndrome, sarcoidosis, dermatomyositis-like syndromes
Endocrine	Type I diabetes mellitus, autoimmune thyroiditis, hypoparathyroidism, adrenal insufficiency, growth failure
Hematologic	Immune thrombocytopenia, autoimmune hemolytic anemia, autoimmune neutropenia, lymphoma
